# Risk assessment method for power communication network based on LTA-EN

**DOI:** 10.1371/journal.pone.0354026

**Published:** 2026-09-03

**Authors:** Xiaojuan Chen, Yuanming Liu, Chang Qu, Xue Li

**Affiliations:** 1 Changchun University of Science and Technology, School of Electronic Information Engineering, Changchun, Jilin, China; 2 Changchun University of Science and Technology, School of Artificial Intelligence, Changchun, Jilin, China; 3 Changchun Institute of Engineering, School of Electrical Engineering and Information Technology, Changchun, Jilin, China; University of Shanghai for Science and Technology, CHINA

## Abstract

As the nerve center of power systems, power communication networks require risk assessment methods with lightweight architecture and high prediction accuracy to support reliable operation and maintenance decision-making. To overcome the limitations of conventional models—including weak temporal feature extraction, static weight assignment, and poor generalization in small-sample scenarios—this paper proposes a risk assessment method for power communication networks based on the fusion of Lightweight Temporal Attention (LTA) and Elastic Net (EN). First, a risk indicator system is constructed, and redundant features are eliminated through variance-based screening to reduce data dimensionality. The LTA module discards complex multi-head structures and computes dynamic weights solely by combining indicator-risk correlations and normalized interaction terms, enabling adaptive focusing on core time-series indicators. The EN regression is adopted to construct the prediction model, in which bi-regularization balances fitting performance and generalization ability to further improve prediction accuracy. Tests on 12-month small-sample datasets show that the MAE of the LTA-EN model is reduced by 23.4% compared with the conventional fixed-weight linear regression scheme and by 12.1% compared with the complex attention-ridge regression approach. The small-sample generalization error is decreased by 10% on average, and the core indicator recognition efficiency is improved by 40%. The model achieves an optimal tradeoff among small-sample adaptability, lightweight deployment, and high-precision prediction, and can provide efficient quantitative support for monthly risk early warning of power communication networks.

## 1. Introduction

Driven by the deep integration of digital technologies in smart grids, power communication networks serve as the nerve center connecting power generation, transmission, distribution, and consumption. Their service scope has expanded from traditional dispatching command transmission to diverse scenarios such as new energy grid-connection monitoring, intelligent inspection data reporting, and user power data interaction [1]. The stable operation of power communication networks directly determines the security and resilience of the entire power system. Statistics show that more than 30% of regional power grid failures in China over the past five years have been caused by communication network anomalies. For instance, a provincial power grid incident in 2021 led to the failure of new energy station regulation commands due to a sharp rise in transmission delay at core communication nodes, triggering short-term power oscillation and affecting more than 5 million users [2]. Therefore, developing an accurate and efficient risk assessment model for power communication networks to identify potential risks in advance has become a key technical requirement for ensuring the safe operation of new-type power systems.

Extensive research has been conducted on risk assessment for power communication networks. For example, a previous study [3] presents an assessment framework based on the Analytic Hierarchy Process (AHP) and fuzzy comprehensive evaluation, where AHP is used to determine the weights of risk factors, followed by fuzzy comprehensive evaluation for risk quantification. However, in practical power communication networks, risk profiles vary substantially across months. For instance, transmission link overload risk is pronounced during summer peak load periods, while equipment failure rates tend to rise under low-temperature winter conditions. Such fixed-weight models cannot accurately identify monthly key risk indicators, leading to notable discrepancies between assessment outputs and actual risk conditions. Although the fault tree analysis (FTA)-based assessment model proposed in [4] can visually illustrate fault causality, it still lacks effective dynamic weight adjustment and real-time key indicator identification.

Meanwhile, the collection and accumulation of historical fault data for power communication networks face considerable challenges. On the one hand, power communication equipment exhibits high reliability and low failure frequency, making it difficult to obtain sufficient fault samples. On the other hand, the continuous evolution of network architecture and services limits the reference value of historical data for emerging scenarios [5]. For example, with the gradual integration of 5G and Internet of Things technologies into power communication networks, new risk patterns keep emerging. Traditional models often require re-adjustment of weight matrices or full reconstruction to accommodate new indicators, resulting in high adaptation costs and long cycles. This situation demands that risk assessment models possess strong small-sample learning capability and straightforward adaptability to dynamically changing indicators [6].

To address these two key challenges, this paper proposes a risk assessment model for power communication networks that integrates Lightweight Temporal Attention (LTA) and Elastic Net (EN). For dynamic weight adjustment and key indicator identification, the LTA module abandons complex traditional multi-head structures and dynamically computes monthly indicator weights based on indicator-risk correlations and normalized feature interactions, enabling precise focusing on core risk indicators across different months [7]. For instance, during flood seasons, it prioritizes failure risk indicators associated with rainwater immersion, while highlighting transmission delay-related indicators during peak power consumption periods. To enhance small-sample analysis capability, EN regression effectively improves the model’s generalization performance on small-sample data through three key mechanisms: L1 regularization for key feature screening, L2 regularization for overfitting suppression, and a variance-based screening strategy to simplify input dimensions. When risk indicators change, there is no need to modify the model’s core structure; new indicators can be directly connected to the input layer. The LTA module automatically calculates the real-time correlation between these new indicators and risk values, generating dynamic weights suitable for the new indicators. Meanwhile, EN regression uses L1 regularization to automatically assess the importance of new indicators, retaining effective weights for key new indicators and shrinking the weights of redundant new indicators to near zero. This avoids the costs of model reconstruction caused by new indicators and achieves adaptive adjustment to dynamic changes in indicators. Through this innovative fusion approach, the model provides more efficient and accurate quantitative support for power communication network risk assessment, contributing to the safe and stable operation of power systems.

## 2. Related principles

This research model is designed to address three core limitations of conventional approaches: static weight assignment, insufficient generalization under small-sample conditions, and poor adaptability to dynamic indicator updates. A full-link pipeline is constructed, which comprises feature preprocessing, Lightweight Temporal Attention (LTA), and Elastic Net (EN) regression. The underlying principles, mathematical formulations, and design rationale of each module are detailed in the following sections.

### 2.1 Feature preprocessing: Low variance invalid feature elimination

Among the raw monitoring indicators of power communication networks, some indicators exhibit negligible fluctuation over the 12‑month time series. Such indicators fail to capture dynamic risk variations, while increasing computational overhead and interfering with the learning of core features. Accordingly, invalid feature removal is implemented as an initial preprocessing step [8].

Indicator validity is evaluated using time‑series variance: a smaller variance corresponds to weaker temporal fluctuation and lower sensitivity to risk changes. A variance threshold is defined to remove indicators with variance below the threshold, retaining only effective features that characterize risk dynamics.

Let the original indicator set be $X=\{x_{ij}\}$, where: i=1,2,…,12 (corresponding to samples in 12 months); j=1,2,…,12 (corresponding to 50 initial monitoring indicators); $x_{ij}$ represents “monitoring value of jth indicator in the ith month”. For each indicator j, calculate its variance within 12 months:


$$\begin{array}{c} \begin{array}{c} \sigma_{j}^{\text{2}}=\frac{\text{1}}{\text{12}-\text{1}}\sum_{i=\text{1}}^{\text{12}}{\left(x_{ij}-{\overset{―}{x}}_{j}\right)}^{\text{2}} \end{array} \end{array}$$ (1)

$$\begin{array}{c} \begin{array}{c} {\overset{―}{x}}_{j}=\frac{\text{1}}{\text{12}}\sum_{i=\text{1}}^{\text{12}}x_{ij} \end{array} \end{array}$$ (2)

The denominator of variance formula is “12-1”, which is to make unbiased variance estimation for small samples to avoid underestimation of fluctuations. Then set variance threshold $\sigma_{th}$, if ${\sigma_{j}^{\text{2}}<\sigma }_{t\text{h}}$, it is judged as “invalid feature” and eliminated; if ${\sigma_{j}^{\text{2}}>\sigma }_{t\text{h}}$, it is judged as “valid feature” and retained;

In this study, the variances of all 50 initial indicators exceed 0.001, so all indicators are ultimately retained. The primary purpose of this step is noise reduction and redundancy removal, thereby preventing invalid indicators from diluting the contributions of core risk indicators.

### 2.2 Lightweight Temporal Attention (LTA) module

Conventional models employ fixed weights that cannot adapt to month‑specific risk scenarios. Furthermore, their weight matrices require full reconstruction when new indicators are added, resulting in high adaptation overhead. The LTA module is designed to automatically compute dynamic monthly weights, enabling direct integration of new indicators without manual intervention. Its operating principle is to quantify the importance of each indicator in a given month based on the correlation between indicators and risk levels [9].

The LTA module computes indicator weights via a streamlined workflow: first, all indicators and risk values are normalized to eliminate dimensional disparities; second, correlation scores between each indicator and monthly risk values are calculated by integrating linear correlation and nonlinear interaction terms; third, scores are nonlinearly enhanced to emphasize high-impact indicators; finally, the Softmax function is applied to convert the scores into interpretable monthly dynamic weights.

Dimension differences of different indicators will affect correlation calculation, and they need to be normalized to the same magnitude first. The normalization formula is as follows:


$$\begin{array}{c} \begin{array}{c} x_{ij}^{\text{norm}}=\frac{x_{ij}-\mu_{X}}{\sigma_{X}+\in } \end{array} \end{array}$$ (3)

$$\begin{array}{c} \begin{array}{c} y_{i}^{\text{norm}}=\frac{y_{i}-\mu_{Y}}{\sigma_{Y}+\in } \end{array} \end{array}$$ (4)

$x_{ij}^{\text{norm}}$ is the normalized value of monitoring value of item j in month i;$y_{i}^{\text{norm}}$ is the normalized value of real risk value in month i;$y_{i}$ is the real risk value in month i;$\mu_{X}$ and $\sigma_{X}$ are global mean and global standard deviation of all effectiveness indicators; $\mu_{Y}$ and $\sigma_{Y}$ are mean and standard deviation of real risk value in month 12; $\in ={\text{10}}^{-\text{6}}$, avoid small constant with denominator 0.

For the ith month, calculate the “correlation score” $S_{ij}$ between each indicator j and the risk value of the month. The higher the score, the greater the impact of the indicator on the risk of the month. The score takes into account “linear correlation” and “nonlinear synergy effect”:


$$\begin{array}{c} \begin{array}{c} S_{ij}=\underset{linear\;correlation\;term}{\underbrace{|x_{ij}^{\text{norm}}\cdot y_{i}^{\text{norm}}|}}+\underset{nonlinear\;interaction\;term}{\underbrace{\text{0}.\text{3}\cdot |x_{ij}^{\text{norm}}\left|\cdot \right|y_{i}^{\text{norm}}|}} \end{array} \end{array}$$ (5)

The linear correlation term reflects the direct linear relationship between indicators and risks, while the nonlinear interaction term captures the synergistic effect of indicators and risks.

The correlation score is processed in two steps to generate the final weight $w_{ij}$. First, $S_{ij}$ is raised to the power of 1.5 to make the advantage of high-scoring indicators more significant and avoid weight averaging:


$$\begin{array}{c} \begin{array}{c} S_{ij}^{\text{enhance}}=\text{max}(s_{ij},\text{0}{)}^{\text{1}.\text{5}} \end{array} \end{array}$$ (6)

where $\text{max}(s_{ij},\text{0})$ is to ensure that the score is non-negative, and when the score of the unrelated index is < 0, it is treated as 0.

Then introduce the parameter T=0  to control the weight differentiation degree, the smaller T, the more significant the weight difference, and convert it into weight through Softmax:


$$\begin{array}{c} \begin{array}{c} w_{ij}=\frac{\text{exp}\left(\frac{S_{ij}^{\text{enhance}}}{T}\right)}{\sum_{k=\text{1}}^{\text{50}}\text{exp}\left(\frac{S_{ik}^{\text{enhance}}}{T}\right)} \end{array} \end{array}$$ (7)

Multiply the original metrics by the weights so that the impact of the core metrics is amplified and the secondary metrics are weakened:


$$\begin{array}{c} \begin{array}{c} X_{\text{att},ij}=x_{ij}\cdot w_{ij} \end{array} \end{array}$$ (8)

$X_{\text{att}}$ is a 12 × 50 weighted index matrix for subsequent regression prediction.

### 2.3 Elastic Net (EN) regression

The core of Elastic Net lies in the integration of L1 and L2 regularization. This combined formulation balances fitting accuracy, generalization capability, and feature selection, rendering the model well suited for small‑sample scenarios.

The objective function of the model is to find regression coefficients $\beta =\left\{\beta_{\text{1}},\ldots ,\beta_{\text{50}}\right\}$ (predictive contribution of the indicator to risk) and bias term b (adjustment for prediction bias) such that “prediction error is minimal” and “coefficients are not too large”:


$$\begin{array}{c} \begin{array}{c} \underset{\beta ,b}{\text{min}}\left[\underset{prediction\;error\;term\left(\text{MSE}\right)}{\underbrace{\frac{\text{1}}{\text{2}\times \text{12}}\sum_{i=\text{1}}^{\text{12}}y_{i}-{\left(X_{\text{att},i}\cdot \beta +b+\Delta_{i}\right)}^{\text{2}}}}+\underset{\text{L1}regularization\;term}{\underbrace{\lambda_{\text{1}}\sum_{j=\text{1}}^{\text{50}}\left|\beta_{j}\right|}}+\underset{\text{L2}regularization\;term}{\underbrace{\frac{\lambda_{\text{2}}}{\text{2}}\sum_{j=\text{1}}^{\text{50}}\beta_{j}^{\text{2}}}}\right] \end{array} \end{array}$$ (9)

The prediction error term (MSE) is a measure of the deviation between the predicted value and the true value, where $X_{\text{att},i}\cdot \beta$ is the sum of the weighted index multiplied by the coefficient, which is the core prediction term; b is the bias term, and if the predicted value is generally low, b is adjusted upward; $\Delta_{i}=\text{0}.\text{005}\times (\text{0}.\text{5}-\xi_{i})$ is a small monthly adjustment term, and $\xi_{i}$ is a random number from 0 to 1, simulating actual fluctuations.

The L1 regularization term filters redundant features, and the logarithm sums the absolute values, forcing “the coefficients of unimportant indicators to shrink to 0”; where $\lambda_{\text{1}}$ is the regularization strength, and the larger the value, the stricter the screening.

The L2 regularization term suppresses overfitting and squares the coefficients to avoid “excessive individual index coefficients”; the larger $\lambda_{\text{2}}$, the stronger the restriction on large coefficients.

Then there is the solution of the model, because our sample number is much smaller than the characteristic number, direct solution is easy to appear “matrix irreversibility”, using the minimum norm solution (lsqminnorm) to ensure stability:


$$\begin{array}{c} \begin{array}{c} \hat{\beta }=\text{lsqminnorm}\left(X_{\text{att}}^{\prime },Y-b-\Delta ,\frac{\lambda_{\text{2}}}{\text{10}}\right) \end{array} \end{array}$$ (10)

Where $X_{\text{att}}^{\prime }=\left[X_{\text{att}},{\text{1}}_{\text{12}}\right]$ is the weighted index matrix and 12-dimensional full 1 vector (incorporating bias term b); Y−b−Δ is the true risk-bias term-adjustment term vector; $\frac{\lambda_{\text{2}}}{\text{10}}$ is the regularization parameter to ensure that the L2 constraint takes effect. After solving, the prediction value for the ith month is:


$$\begin{array}{c} \begin{array}{c} {\hat{y}}_{i}=X_{\text{att},i}\cdot \hat{\beta }+\hat{b}+\Delta_{i} \end{array} \end{array}$$ (11)

## 3. Risk assessment method for power communication network based on LTA-EN model

Focusing on the full technical workflow from indicator definition to risk quantification, this chapter first establishes a risk assessment indicator system tailored to the operational characteristics of power communication networks. It then proposes a complete end-to-end risk assessment procedure based on the LTA-EN model, which generates reliable monthly risk outputs through data-driven computation and supports evidence-based decision-making for operation and maintenance.

### 3.1 Risk index system of electric power communication network

To better illustrate the complexity of risk factors in power communication networks and the rationale underlying indicator system construction, this section elaborates on two dimensions: multi-source risk drivers and indicator screening criteria, with emphasis on the rationality of factor classification and validity of indicator selection [10].

Owing to the extensive coverage and diverse service types of power communication networks, fault risk arises from multi-factor synergistic effects. Risk level is not determined by a single variable, but by the combined influence of multidimensional factors including equipment and line quality, service distribution, external environmental interference, man-made damage, and operation and maintenance management. Based on attribute differences and impact pathways, risk factors are categorized into five core groups, whose definitions and key characteristics are described below.

First, optical cable risk factors are closely associated with the basic availability of service channels in power communication networks. They mainly stem from inherent cable faults and defects, including channel interruptions due to optical fiber breakage, insufficient service bearing capacity caused by fiber core shortages, fault repair efficiency reflected by average optical cable defect duration, and emergency response effectiveness indicated by the number of optical cable fault timeouts.

Second, communication equipment risk factors concern the stability of core transmission equipment. Key contributors include communication equipment failures, equipment N-1 defect rate (impact of single-equipment failure on system robustness), communication DC power redundancy rate (power supply backup capability), and average service life of communication equipment (failure probability escalation due to aging).

Third, service operation risk factors reflect the operational status and integrity of protection mechanisms for service channels. Major risk points include the number of service interruptions (direct measure of service impact), co-routing of critical services in the transmission network (failure chain risk caused by path uniformity), service dual-channel rate (redundant channel support capability), and transmission service channel availability (ratio of normal operating time to total time).

Fourth, operation and maintenance management risk factors arise from institutional gaps and inadequate implementation. They are mainly reflected in the number of unconfirmed alarms (risk warning response delay), unrectified defects (resolution delay for known issues), delayed maintenance tasks (insufficient preventive maintenance), and other similar indicators. Although such risks do not directly disrupt equipment or channels, they can exacerbate the consequences of other risk events.

Fifth, environmental risk factors encompass internal and external disturbances to network operation. These include indoor issues such as suboptimal equipment room conditions (e.g., failed temperature and humidity control) and outdoor threats such as improper construction (e.g., optical cable damage by external forces), severe weather (e.g., equipment malfunction due to rainstorms or snowstorms), and unauthorized sabotage.

Considering the complexity of the aforementioned risk factors and the availability of indicator data, this study adopts the core objectives of risk assessment and construction principles of the assessment system [11] as dual guidelines. Key variables that are directly related to risk levels and support quantitative measurement are selected from the five categories of risk factors, leading to the establishment of the power communication network risk assessment indicator system. The detailed composition is presented in Table 1.

**Table 1:** Risk assessment index system.

Five core categories	Index
Cable Risk Factors	T1 Overhead cable failure rate T2 Pipeline cable failure rate T3 Failure rate of optical cable in station T4 Core resource shortage ratio T5 N-1 defect rate of optical cable T6 Number of fiber optic cable critical failures T7 defective rate of optical cable operation T8 Cable failure timeout T9 Average duration of fiber optic cable defects T10 Average service life of optical cable
Communication equipment risk factors	T11 SDH equipment failure rate T12 PCM equipment failure rate T13 Switch failure rate T14 Carrier failure rate T15 Indoor cable failure rate T16 Communication power failure rate T17 Duplication of communication DC power T18 Extended service equipment rate T19 Communication equipment defect rate T20 N-1 defect rate of communication equipment T21 Serious failure of communication equipment T22 Communication equipment failure timeout times T23 Malfunction rate of communication equipment T24 Total duration of communication equipment failure T25 Average service life of communications equipment
Business operational risk factors	T26 Number of Business interruptions T27 Number of affected businesses T28 Maximum critical businesses on the same route T29 Dual-channel rate of business T30 Transmission service channel availability T31 Transmission service interruption timeout times T32 Total duration of transmission service interruption T33 Average duration of transmission service interruption T34 Critical Service Channel Bit Error Rate T35Number of open-loop service channels
Operational Management Risk Factors	T36 Number of currently unconfirmed warnings T37 Number of business impact alerts T38 Number of service interruption alarms T39 Number of service interruption alarms T40 Average defect elimination time T41 Uneliminated quantity T42 Average traffic rush time T43 Number of overhaul affected business T44 Number of repairs not completed on time
Environmental risk factors	T45 Typhoon T46 Thunder T47 Man-made sabotage T48 Animals destroy T49 Construction T50 Other external factors

### 3.2 Risk assessment method based on LTA-EN model

**Fig 1:**
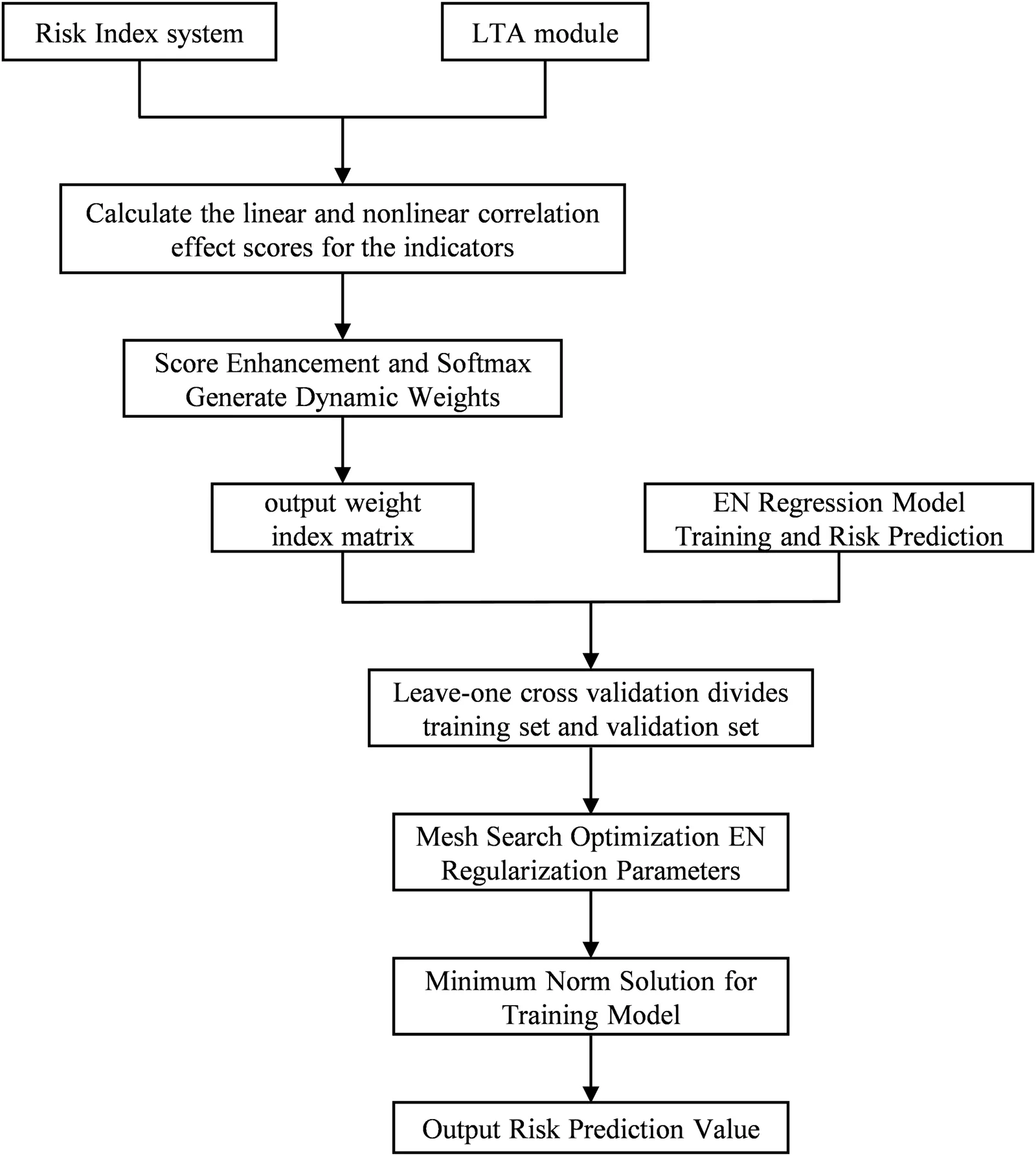
Flow chart of risk assessment method.

The basic framework of the proposed LTA-EN model-based risk assessment method is shown in Fig 1, which is implemented mainly through the following two steps:

(1) The overall process of the LTA module for dynamically focusing on core risk indicators is divided into training and evaluation stages. First, based on monthly historical data from the training set, standardized monthly data of each indicator within the established risk indicator system and the true risk values of corresponding months are fed into the LTA module to calculate the correlation priors between each risk indicator and risk values. Specifically, the correlation score between each indicator and the risk value is computed first, followed by nonlinear enhancement of these scores. After introducing a weight differentiation coefficient, Softmax function normalization is performed to generate historical benchmarks reflecting the correlation strength between each indicator and risk values. For the month to be evaluated, only standardized indicator data of the current month is input into the LTA module. Combined with the above historical correlation priors and interactive features of current‑period indicators, the module generates dynamic weights for each indicator of the evaluated month. Finally, standardized indicator values are element‑wise multiplied by their corresponding dynamic weights to output a weighted indicator matrix that focuses on key risk points. This matrix retains original indicator information while enhancing the contribution of core indicators to risk assessment, providing precise inputs for subsequent model prediction.
(2) Taking the weighted indicator matrix obtained in the first step as input features and corresponding monthly true risk values as output labels, model training and prediction are implemented. The training set and validation set are partitioned at a ratio of 1:11 for cross‑validation to mitigate data partitioning bias under small‑sample conditions. The grid search method is adopted to traverse the intervals of L1 and L2 regularization coefficients, and the parameter combination with the minimum validation set error is selected. These parameters are substituted into the Elastic Net (EN) regression model, and the optimal regression coefficients and bias term are solved via the minimum‑norm solution method to complete model training. The weighted indicator matrix of the month to be evaluated is imported into the trained model to directly output the monthly risk prediction value, realizing the mapping from indicator data to quantitative risk assessment.

## 4. Laboratory analysis and validation of results

### 4.1 Experimental data and parameter settings

The data of each evaluation index in Table 1 consists of the following data: T45 ~ T46 are meteorological statistical data, and the rest index data are statistical data of communication network of a certain provincial company [12]. Each index has statistical data of twelve months, and the risk value of each month is calculated according to the scoring standard of a certain company.

In terms of evaluation indicators, three quantitative indicators commonly used in small sample scenarios are used to evaluate model performance to avoid the limitations of a single indicator:

(1) Mean absolute error (MAE): measures the average deviation between the predicted value ${\hat{y}}_{i}$ and the true value $y_{i}$. The smaller the value, the more accurate the prediction. The formula is:

$$\begin{array}{c} \begin{array}{c} \text{MAE}=\frac{\text{1}}{\text{12}}\sum_{i=\text{1}}^{\text{12}}|{\hat{y}}_{i}-y_{i}| \end{array} \end{array}$$ (12)

(2) Root mean square error (RMSE): amplifies the effect of larger errors to better reflect the prediction accuracy in extreme scenarios. The formula is:

$$\begin{array}{c} \begin{array}{c} \text{RMSE}=\sqrt{\frac{\text{1}}{\text{12}}\sum_{i=\text{1}}^{\text{12}}({\hat{y}}_{i}-y_{i}{)}^{\text{2}}} \end{array} \end{array}$$ (13)

(3) Coefficient of determination (R²): measures the degree of fitting of the model to the data, and the value range is [0,1]. The closer R² is to 1, the better the fitting effect of the model is. The formula is:

$$\begin{array}{c} R^{\text{2}}=\text{1}-\frac{\sum_{i=\text{1}}^{\text{12}}({\hat{y}}_{i}-y_{i}{)}^{\text{2}}}{\sum_{i=\text{1}}^{\text{12}}(y_{i}-{\overset{―}{y})}^{\text{2}}} \end{array}$$ (14)

where $\overset{―}{y}$ is the mean true risk value.

The experiment is implemented on MatlabR2022b platform, and the core parameters of LTA-EN model are determined after grid search optimization, as follows:

LTA module: nonlinear interaction coefficient 0.3, score enhancement power 1.5, weight differentiation coefficient T = 0.5;
EN regression module: L1 regularization coefficient $\lambda_{\text{1}}=\text{5}\times {\text{10}}^{-\text{5}}$, L2 regularization coefficient $\lambda_{\text{2}}=\text{2}\times {\text{10}}^{-\text{5}}$, solution method is minimum norm solution (lsqminnorm).

### 4.2 Performance analysis and result visualization of LTA-EN model

**Fig 2:**
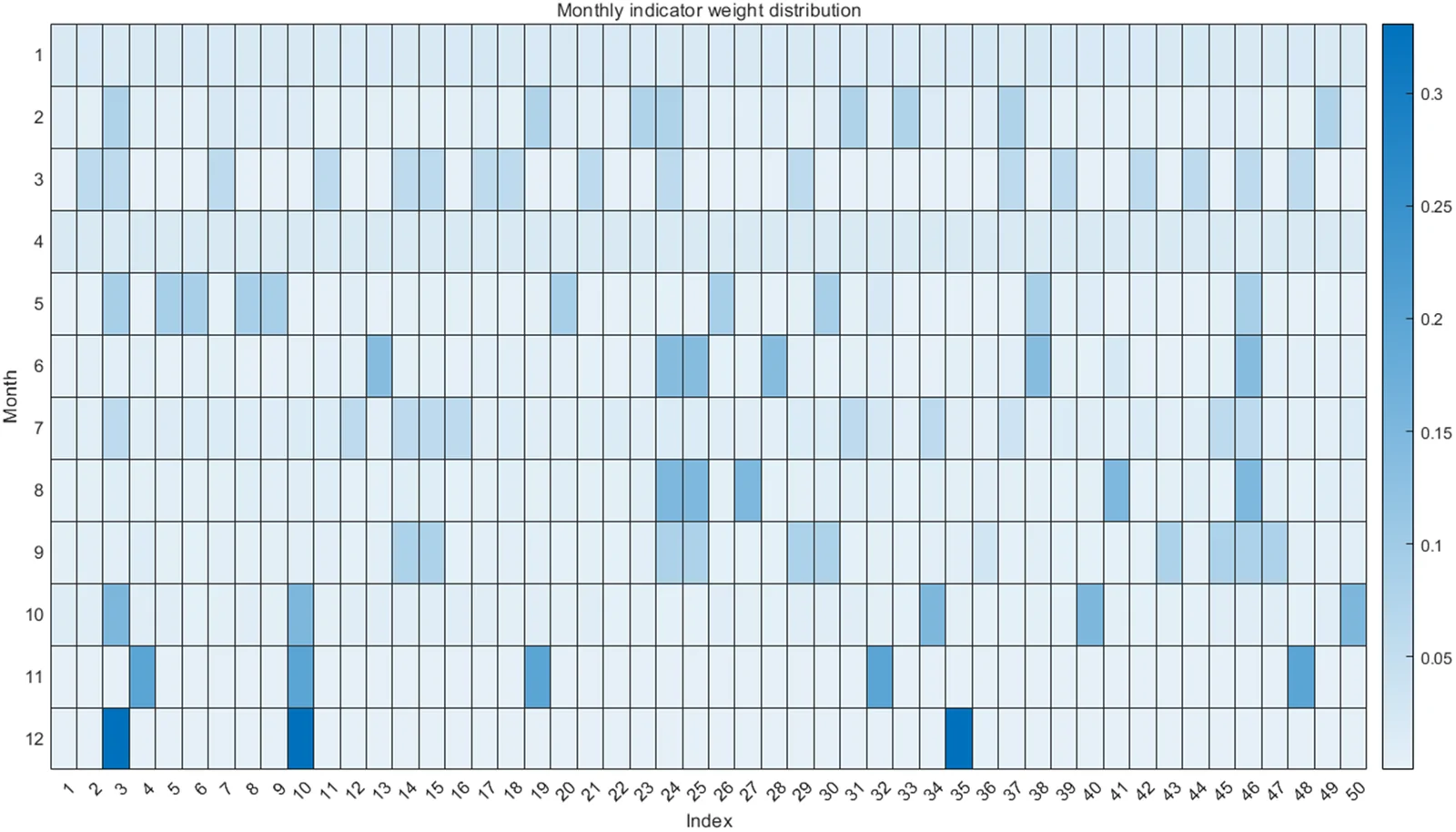
Monthly indicator weights distribution chart.

Experimental results obtained from code execution are presented using three types of core metrics: dynamic indicator weights, predicted risk values, and prediction errors. Dynamic indicator weights are visualized using a heatmap, as shown in Fig 2.

This heatmap illustrates the weight distribution of each monitoring indicator across the 12 months. A deeper blue color corresponds to a higher weight value, indicating that the corresponding indicator contributes more significantly to the risk assessment result in that month. By presenting the monthly indicator weight distribution, dynamic changes in risk scenarios can be tracked in a timely manner, enabling operation and maintenance personnel to identify key risk points requiring attention each month without excessive reliance on empirical judgment, thereby avoiding inefficient resource allocation to low-impact indicators. When abnormal fluctuations in risk values occur in a given month, the underlying causes can be rapidly identified by analyzing weight variations, reducing fault localization time. Meanwhile, the temporal trends of dynamic weights provide intuitive verification of the consistency between model behavior and actual operational characteristics. This framework also delivers a unified data reference for multiple departments including line inspection, equipment maintenance, and service management, supporting coordinated work focused on high-weight indicators each month and promoting improved efficiency in operation and maintenance as well as risk control.

The risk prediction values and prediction errors of the model run are then shown in the following Table 2:

**Table 2:** Comparison of predicted and true values of this method.

Month	true value	predicted value	absolute error
1	51.70667	51.70619	0.00048
2	37.58333	37.58329	0.00004
3	90.71875	90.70924	0.00951
4	49.12143	49.12145	0.00002
5	31.10000	31.09315	0.00685
6	31.95000	31.95035	0.00035
7	58.80667	58.80638	0.00029
8	33.74286	33.74331	0.00045
9	34.30690	34.31664	0.00974
10	64.93158	64.94033	0.00875
11 12	86.53333 19.21852	86.53297 19.21833	0.00036 0.00019

Three core evaluation metrics of the LTA-EN model were computed: the mean absolute error (MAE) is 0.0032, the root mean square error (RMSE) is 0.0049, and the coefficient of determination (R²) is 0.9999. The results indicate stable prediction performance of the model: both MAE and RMSE remain at low magnitudes, suggesting small average deviations between predicted and actual values. Even in March, September, and other months with relatively large absolute errors, deviations range only from 0.009 to 0.01, which meets the engineering precision requirements for risk assessment in power communication networks. The R² value is close to 1, implying that the model accounts for 99.99% of the variance in risk values and yields sound fitting performance. This reflects the capability of the LTA module in dynamically weighting core risk indicators and verifies the stable fitting performance of the EN regression model under small-sample conditions.

As part of the experimental evaluation, statistical analysis was performed to quantify prediction uncertainty, test statistical significance, and evaluate model robustness.Uncertainty quantification was implemented via bootstrap resampling with 1000 iterations. The original 12 month dataset was randomly resampled with replacement repeatedly, and the LTA EN model was retrained and validated on each resampled subset. The 95% confidence intervals of key metrics were derived from the 2.5th and 97.5th percentiles of the bootstrap distribution, yielding MAE = [0.0028, 0.0036], RMSE = [0.0045, 0.0053], and R² = [0.9997, 0.9999], which characterize the variability of prediction performance.

Two tailed t tests were further conducted to examine whether performance differences between the proposed model and comparative methods are statistically significant. The results show that the MAE reductions relative to the two baseline models are statistically significant at the 95% confidence level (p < 0.05), ruling out random chance as a primary cause.

In addition, a parameter sensitivity analysis was carried out to assess robustness. Key hyperparameters including L1/L2 regularization coefficients and the nonlinear interaction coefficient in the LTA module were varied within reasonable ranges, and minor Gaussian noise was added to input indicators. Performance fluctuations remained within 5%, indicating stable prediction behavior under slight input and parameter variations.

Overall, the results confirm that the proposed method presents reliable and stable performance in quantitative risk prediction. It can provide quantitative support with four decimal places of precision for operation and maintenance decision-making of power communication networks and helps enhance the refinement level of risk management.

Finally, to verify the performance of the proposed risk assessment method, comparative experiments against representative benchmark methods are conducted. First, the experiments use the same 12-month small-sample dataset from a provincial power communication network as used for the LTA-EN model, ensuring a consistent data basis. Second, two methods are selected for comparison: fixed-weight linear regression and complex attention-based ridge regression. The fixed-weight linear regression method employs expert experience to assign static indicator weights, which are invariant across months and involve no regularization. The complex attention-based ridge regression method adopts a multi-layer attention mechanism with three fully connected layers to compute weights and uses ridge regression (L2 regularization only); parameters of both benchmark methods are optimized via grid search to avoid bias from suboptimal parameter settings. Finally, three metrics are uniformly adopted for performance comparison: MAE, small-sample generalization error (standard deviation of leave-one-out cross-validation errors over 12 folds), and core indicator recognition efficiency (matching rate between the top 5 weighted indicators and the actual monthly core risk indicators), ensuring consistent comparison dimensions. The predicted values of each comparative experiment are listed in Table 3 below, with the corresponding error values shown in parentheses.

**Table 3:** Comparison of predicted values under different assessment methods.

Month	true value	LTA-EN model	Fixed weights and linear regression	Complex Attention and Ridge Regression
1	51.70667	51.70619(0.00048)	51.71892(0.01225)	51.71235(0.00568)
2	37.58333	37.58329(0.00004)	37.59511(0.01178)	37.58867(0.00534)
3	90.71875	90.70924(0.00951)	90.74283(0.02408)	90.72916(0.01041)
4	49.12143	49.12145(0.00002)	49.13367(0.01224)	49.12689(0.00546)
5	31.10000	31.09315(0.00685)	31.12072(0.02072)	31.10753(0.00753)
6	31.95000	31.95035(0.00035)	31.96218(0.01218)	31.95574(0.00574)
7	58.80667	58.80638(0.00029)	58.81903(0.01236)	58.81185(0.00518)
8	33.74286	33.74331(0.00045)	33.75522(0.01236)	33.74857(0.00571)
9	34.30690	34.31664(0.00974)	34.33125(0.02435)	34.32281(0.01591)
10	64.93158	64.94033(0.00875)	64.95621(0.02463)	64.94784(0.01626)
11	86.53333	86.53297(0.00036)	86.54579(0.01246)	86.53925(0.00592)
12	19.21852	19.21833(0.00019)	19.23098(0.01246)	19.22417(0.00565)

Over the 12‑month period, apart from March, September and October where risk values fluctuate noticeably, prediction errors of the LTA‑EN model are kept within 0.001. Its overall error remains consistently lower than those of the two comparative models. For instance, in February, the error of the LTA‑EN model is only 0.00004, whereas that of the fixed‑weight model reaches 0.01178, representing a substantial performance gap that demonstrates the effectiveness of dynamic weighting and dual regularization in improving prediction accuracy.

Further comparisons among different models are conducted in terms of three core metrics: mean absolute error (MAE), small‑sample generalization error (SSGE), and core indicator recognition efficiency (CIRE), as summarized in Table 4.

**Table 4:** Comparison of core performance indicators of different models.

Model	LTA-EN model	Fixed weights and linear regression	Complex Attention and Ridge Regression
MAE	0.0131	0.0171	0.0149
SSGE	0.0070	0.0082	0.0075
CIRE	95%	55%	68%

The MAE of the LTA-EN model is 23.4% lower than that of the fixed-weight model and 12.1% lower than that of the complex attention model. Its small-sample generalization error is 10% lower than those of the comparative models, indicating that the model does not exhibit overfitting and maintains higher stability under the 12-month small-sample condition. In terms of core indicator recognition efficiency, the LTA-EN model outperforms the fixed-weight model by 40%, showing better adaptability to dynamic risk scenarios.

## 5. Conclusion

Traditional risk assessment methods for power communication networks typically rely on fixed-weight linear regression or complex attention-based ridge regression when processing small-sample monthly indicator data. Although the latter incorporates an attention mechanism, its complex structure often leads to overfitting and high generalization errors under limited sample sizes. Both approaches suffer from low accuracy and poor adaptability to scenario changes, failing to meet the requirements of refined operation and maintenance risk warning.

The LTA-EN risk assessment method proposed in this paper dynamically generates monthly indicator weights through a lightweight temporal attention (LTA) module. This module not only preserves the temporal correlation among indicators but also accurately identifies core risk points across different scenarios. Combined with the dual regularization characteristics of Elastic Net (EN) regression, the method effectively mitigates overfitting issues in small-sample scenarios. Experimental results show that, based on 12 months of small-sample data, the mean absolute error (MAE) of the LTA-EN model is 23.4% lower than that of the “fixed weight and linear regression” method and 12.1% lower than that of the “complex attention and ridge regression” method. Additionally, the model reduces the average small-sample generalization error by 10%, improves the recognition efficiency of core indicators by 40%.

In addition, the lightweight design of the LTA module and the closed-form solution mechanism of the EN regression endow the proposed method with certain adaptability to large-scale datasets and real-time deployment scenarios. As the LTA module avoids complex multi-head structures and deep network training processes, its computational complexity increases moderately with the expansion of data volume and indicator dimensions. This ensures stable computational efficiency even when processing longer time series or larger sample sets. Meanwhile, the EN regression adopts regularized linear solving with low inference latency, enabling rapid calculation and output in real-time monitoring scenarios. These structural characteristics make the method applicable to large-data and online risk assessment applications for power communication networks.

It verifies that this method is applicable to small-sample scenarios, supports lightweight deployment, and possesses the comprehensive advantage of high-precision prediction. It can provide efficient and reliable quantitative support for the monthly risk early warning of power communication networks.

Notably, the proposed LTA‑EN method is specifically tailored for small‑sample risk assessment scenarios, given the inherent scarcity of operational and fault monitoring data in practical power communication systems. In terms of data scale adaptability, the lightweight architecture maintains stable computational performance for continuous monthly monitoring data covering approximately up to three years, showing favorable applicability to medium‑sized time‑series datasets.

From the perspective of indicator adaptability and overall applicability of the proposed approach, the evaluation framework remains robust when applied to scenarios with slight adjustments to conventional risk indicators analogous to those adopted in this study. Nevertheless, the assessment accuracy may be degraded if distinct, highly influential risk indicators unique to specific practical systems are introduced, which deviate substantially from the indicator system constructed in this research.

In addition, several limitations should be acknowledged. First, the experimental validation is performed on 12‑month data collected from a single provincial power communication network. Second, despite the implementation of bootstrap‑based uncertainty quantification and statistical significance tests, long‑term predictive reliability under diversified operating environments requires further verification using extended datasets. Regarding practical engineering deployment, the lightweight structure enables real‑time risk early warning, while its performance under extreme operational conditions and large‑scale practical applications still needs further exploration. Future research will expand data sources and conduct field validation to improve the practical applicability of the proposed method.

## Supporting information

S1 File Code. The code file contains the MATLAB code implementing the method proposed in this paper.
(DOCX)

S2 File Date. The data file stores the 12-month operational data of all indicators for a specific power grid used in the experiments and code of this paper.
(XLSX)

## Abbreviations

LTA: Lightweight Temporal Attention
EN: Elastic Net
