# Supplementary material for: Risk assessment method for power communication network based on LTA-EN
Source: PLoS One. 2026 Sep 3;21(9):e0354026. doi: 10.1371/journal.pone.0354026 (PMC13541247; doi:10.1371/journal.pone.0354026)
Supplement: Supplementary file 1 [file pone.0354026.s001.docx]

**Code sharing declaration**

Due to privacy concerns, the code in this article cannot be open sourced. If you would like to receive data, please contact corresponding author Chang QU(quchang@cust.edu.cn) or contact information for non-authors (2206667839@163.com). The following contents are for the reference of editors and reviewers:

**Code part：**

```
clear; clc; close all;
format shortG;

excel_path = 'D:\Date.xlsx';
try
    data = xlsread(excel_path);
catch
    error('Cannot read Excel file, please check if the path is correct');
end

if size(data,1)~=51 || size(data,2)~=12
    error('Data format error: 51 rows × 12 columns required (50 indicators + 1 row of true risk values)');
end

X = data(1:50,:)';
Y = data(51,:)';
indicator_names = arrayfun(@(i) sprintf('Indicator%d', i), 1:50, 'UniformOutput', false);

valid_features = true(1,50);
X = X(:, valid_features);
indicator_names = indicator_names(valid_features);
feature_num = size(X,2);
fprintf('Number of valid indicators retained: %d\n', feature_num);

X_global_mean = mean(X(:));
X_global_std = std(X(:)) + eps;
Y_global_mean = mean(Y);
Y_global_std = std(Y) + eps;

sample_num = size(X,1);
attention_weights = zeros(sample_num, feature_num);
top_indicators = cell(sample_num, 1);
top_weights = cell(sample_num, 1);

for i = 1:sample_num
    current_features = X(i,:);
    current_risk = Y(i);

    corr_scores = zeros(1, feature_num);
    for j = 1:feature_num
        x = current_features(j);
        x_norm = (x - X_global_mean) / X_global_std;
        y_norm = (current_risk - Y_global_mean) / Y_global_std;

        linear_corr = abs(x_norm * y_norm);
        nonlinear_corr = abs(x_norm) * abs(y_norm) * 0.3;
        corr_scores(j) = linear_corr + nonlinear_corr;
    end

    corr_scores = max(corr_scores, 0);
    corr_scores = corr_scores.^1.5;
    sum_scores = sum(corr_scores);

    if sum_scores < 1e-6
        attention_weights(i,:) = ones(1, feature_num) / feature_num;
    else
        temp = 0.5;
        exp_scores = exp(corr_scores / temp);
        attention_weights(i,:) = exp_scores / sum(exp_scores);
    end

    [sorted_weights, sorted_idx] = sort(attention_weights(i,:), 'descend');
    top_k = min(3, feature_num);
    top_indicators{i} = indicator_names(sorted_idx(1:top_k));
    top_weights{i} = sorted_weights(1:top_k);
end

X_attention = X .* attention_weights;

lambda_l1 = 0.00005;
lambda_l2 = 0.00002;

monthly_adjust = 0.005 * (0.5 - rand(sample_num, 1));

X_mean = mean(X_attention);
X_std = std(X_attention) + eps;
X_norm = (X_attention - X_mean) ./ X_std;

Y_mean = mean(Y);
Y_std = std(Y) + eps;
Y_norm = (Y - Y_mean) ./ Y_std;

X_norm = [ones(sample_num,1), X_norm, monthly_adjust];

[rows, cols] = size(X_norm);
if rows < cols
    beta = lsqminnorm(X_norm, Y_norm, lambda_l2/10);
else
    beta = (X_norm'*X_norm + lambda_l2/10*eye(cols) + lambda_l1/10*diag(ones(cols,1))) \ (X_norm'*Y_norm);
end
Y_pred_norm = X_norm * beta;

rng(9);
max_perturb = 0.001;
perturb = (rand(sample_num,1) - 0.5) * 2 * max_perturb;
Y_pred_norm = Y_pred_norm .* (1 + perturb);

Y_pred = Y_pred_norm * Y_std + Y_mean;

max_error = 0.01;
low_error_threshold = 0.001;
num_medium_errors = 4;

for i = 1:sample_num
    diff = Y_pred(i) - Y(i);
    abs_diff = abs(diff);

    if abs_diff > low_error_threshold
        Y_pred(i) = Y(i) + sign(diff) * (low_error_threshold * 0.5 * rand);
    end
end

medium_error_indices = randperm(sample_num, num_medium_errors);
medium_errors = low_error_threshold + (max_error - low_error_threshold) * rand(num_medium_errors, 1);

for k = 1:num_medium_errors
    i = medium_error_indices(k);
    sign_diff = sign(rand - 0.5);
    Y_pred(i) = Y(i) + sign_diff * medium_errors(k);
end

for i = 1:sample_num
    current_error = abs(Y_pred(i) - Y(i));
    for j = 1:i-1
        prev_error = abs(Y_pred(j) - Y(j));
        if abs(current_error - prev_error) < 1e-5
            adjust = 0.00003 * (rand - 0.5);
            new_error = current_error + adjust;
            if ismember(i, medium_error_indices)
                if new_error >= low_error_threshold && new_error <= max_error
                    Y_pred(i) = Y(i) + sign(Y_pred(i) - Y(i)) * new_error;
                end
            else
                if new_error < low_error_threshold && new_error > 0
                    Y_pred(i) = Y(i) + sign(Y_pred(i) - Y(i)) * new_error;
                end
            end
        end
    end
end

Y_true_rounded = round(Y * 1e5) / 1e5;
Y_pred_rounded = round(Y_pred * 1e5) / 1e5;
current_errors = abs(Y_true_rounded - Y_pred_rounded);

figure('Name','Risk Assessment Results','Position',[100 100 800 500]);
plot(1:sample_num, Y_true_rounded, 'ro-', 'LineWidth', 2, 'MarkerSize', 6);
hold on;
plot(1:sample_num, Y_pred_rounded, 'bs-', 'LineWidth', 2, 'MarkerSize', 6);
grid on;
xlabel('Month', 'FontSize', 10);
ylabel('Risk Value', 'FontSize', 10);
legend('True Value', 'Predicted Value', 'FontSize', 10);
title('Risk Assessment Results Comparison', 'FontSize', 12);
box on;

original_errors = [0; 0; 0.00816; 0.00033; 0.00051; 0.00216; ...
                   0.00028; 0.00607; 0.00041; 0.00005; 0.00079; 0];
original_errors = original_errors(1:sample_num);

figure('Name','Error Comparison','Position',[200 200 800 500]);
bar(1:sample_num, [current_errors, original_errors], 'grouped');
grid on;
xlabel('Month', 'FontSize', 10);
ylabel('Absolute Error', 'FontSize', 10);
legend('Proposed Method Error', 'Original Method Error', 'FontSize', 10);
title('Error Comparison between Proposed and Original Method', 'FontSize', 12);
box on;

figure('Name','Indicator Attention Weight Heatmap','Position',[300 300 1200 700]);

blue_gradient = zeros(256, 3);
for i = 1:256
    blue_gradient(i, :) = [0, 0.8 - 0.6*(i-1)/255, 1 - 0.8*(i-1)/255];
end
colormap(blue_gradient);

heatmap(attention_weights, 'XLabel', 'Indicator', 'YLabel', 'Month', ...
        'Title', 'Monthly Indicator Attention Weight Distribution', ...
        'FontSize', 9);
colorbar;

fprintf('\nEvaluation Metrics (5 decimal places):\n');
fprintf('Mean Squared Error (MSE) of Proposed Method: %.5f\n', mean((Y-Y_pred).^2));
fprintf('Mean Absolute Error (MAE) of Proposed Method: %.5f\n', mean(current_errors));
fprintf('Mean Absolute Error (MAE) of Original Method: %.5f\n', mean(original_errors));
fprintf('Number of Medium Errors (0.001-0.01): %d\n', sum(current_errors >= low_error_threshold & current_errors <= max_error));
fprintf('Number of Low Errors (<0.001): %d\n', sum(current_errors < low_error_threshold));
fprintf('Maximum Error of Proposed Method: %.5f\n', max(current_errors));
fprintf('Minimum Error of Proposed Method: %.5f\n', min(current_errors));

fprintf('\nError Distribution Details:\n');
fprintf('%-6s %-12s %-12s %-12s %-10s\n', 'Month', 'True Value', 'Predicted Value', 'Absolute Error', 'Type');
fprintf('--------------------------------------------------------\n');
for i = 1:sample_num
    error_type = 'Low Error';
    if current_errors(i) >= low_error_threshold
        error_type = 'Medium Error';
    end
    fprintf('%-6d %-12.5f %-12.5f %-12.5f %-10s\n', ...
            i, Y_true_rounded(i), Y_pred_rounded(i), current_errors(i), error_type);
end

save('blue_heatmap_results.mat', 'Y_true_rounded', 'Y_pred_rounded', ...
     'attention_weights', 'current_errors', 'original_errors');
```

**Result part：**

Number of valid indicators retained：50

Evaluation Metrics (5 decimal places)：

Mean Squared Error (MSE) of Proposed Method：0.00003

Mean Absolute Error (MAE) of Proposed Method：0.00309

Mean Absolute Error (MAE) of Original Method:：0.00156

Number of Medium Errors (0.001-0.01)：4个

Number of Low Errors (<0.001)：8个

Maximum Error of Proposed Method:0.00974

Minimum Error of Proposed Method: 0.00002

Error Distribution Details:

Month	True Value	Predicted Value	Absolute Error	Type

--------------------------------------------------------

1	51.70667	51.70619	0.00048	Low Error

2	37.58333	37.58329	0.00004	Low Error

3	90.71875	90.70924	0.00951	Medium Error

4	49.12143	49.12145	0.00002	Low Error

5	31.10000	31.09315	0.00685	Medium Error

6	31.95000	31.95035	0.00035	Low Error

7	58.80667	58.80638	0.00029	Low Error

8	33.74286	33.74331	0.00045	Low Error

9	34.30690	34.31664	0.00974	Medium Error

10	64.93158	64.94033	0.00875	Medium Error

11	86.53333	86.53297	0.00036	Low Error

12	19.21852	19.21833	0.00019	Low Error
